# Integration of Immune Cell Signatures and Diagnostic Gene Markers in Pancreatitis: A Comprehensive Study on Therapeutic Targets and Predictive Diagnosis

**DOI:** 10.1155/humu/7694723

**Published:** 2025-08-01

**Authors:** Qianyu Xie, Birong Liu, Xiao Yu, Xiang Wei, Qiangsheng Xiao

**Affiliations:** ^1^Department of Interventional Vascular Surgery, Changsha Fourth Hospital, Changsha, China; ^2^Department of Hematology, The Third Xiangya Hospital of Central South University, Changsha, China; ^3^Department of Hepatobiliary and Pancreatic Surgery, The Third Xiangya Hospital of Central South University, Changsha, China

**Keywords:** bioinformatics, hypoxia, immune infiltration, machine learning, pancreatitis

## Abstract

Pancreatitis is a severe and increasingly prevalent disease that affects the digestive system. Early detection and accurate diagnosis of this condition are crucial for reducing mortality rates and improving patient outcomes. Therefore, the development of novel diagnostic markers is essential for enhancing clinical management and advancing the understanding of pancreatitis. The initial phase involved applying the ssGSEA method to extract hypoxia scores from these samples. Subsequently, a thorough differential expression analysis was performed, complemented by functional assessments and various machine learning techniques designed to pinpoint critical diagnostic genes relevant to pancreatitis. From this, a robust diagnostic model was constructed and validated using a series of machine learning strategies. To further validate our results, molecular docking studies were conducted to determine the binding affinities between the identified markers and standard medications such as omeprazole and lansoprazole. Additionally, the ssGSEA methodology was leveraged to compute immune cell scores within the pancreatitis samples, thus enriching the analysis of the relationships between significant diagnostic genes and various immune cell types. Finally, the experiments of ELISA and qRT-PCR were used to verify the expression of key target genes. Through WGCNA, we identified a total of 50 genes associated with hypoxic conditions within the pancreatitis samples. Further investigations, including differential expression analysis and machine learning techniques, revealed six significant diagnostic markers for pancreatitis: RAP1GDS1, TOP2A, ADK, POLL, CD44, and CD4. The diagnostic model we developed exhibited a high accuracy level in predicting pancreatitis onset, while molecular docking analyses indicated that these six key diagnostic genes hold promise as drug targets. Moreover, the ssGSEA algorithm confirmed the relationships between these diagnostic markers and a range of immune cell populations. Ultimately, the expression levels of the identified key genes were rigorously validated through experimental techniques, reinforcing the credibility of our findings.

## 1. Introduction

Pancreatitis is a common and severe condition affecting the digestive system, characterized by an increasing incidence rate, particularly among individuals with acute and chronic forms of the disease [1]. Early diagnosis is crucial for reducing patient mortality and improving prognoses [2]. However, traditional diagnostic markers like serum amylase and lipase have notable limitations; they can be influenced by factors such as hyperlipidemia and hepatobiliary diseases, leading to potential false negatives or positives [3]. Furthermore, these conventional markers often do not adequately represent the extent of pancreatic damage in the early disease stages. Thus, the development of more specific and sensitive diagnostic markers has become increasingly vital. Accurate and timely diagnosis not only assists healthcare providers in formulating appropriate treatment plans but also facilitates early intervention strategies that minimize complications and enhance patients' quality of life [4–7]. With ongoing advancements in medical technology, there is a growing emphasis on personalized medicine. The identification of novel diagnostic markers will support assessment across diverse patient populations, thereby bolstering personalized medicine initiatives. Additionally, research into new diagnostic markers enhances clinical diagnosis accuracy and offers insights into pancreatitis pathogenesis, fostering synergy between basic research and clinical application.

Hypoxia significantly influences the pathophysiology of pancreatitis, particularly in both acute and chronic disease contexts. The interplay between hypoxia and pancreatitis involves multiple cellular and molecular mechanisms that contribute to inflammation, tissue injury, and disease progression. In acute pancreatitis (AP), hypoxia can result from vascular damage and edema, which disrupts blood flow and oxygen delivery to pancreatic tissues. This oxygen deprivation can intensify the inflammatory response, resulting in further tissue damage and necrosis. The activation of hypoxia-inducible factors (HIFs), especially HIF-1*α*, is a key response to hypoxic conditions. HIF-1*α* stabilizes under low oxygen levels, activating transcription of genes crucial for metabolic adaptation, angiogenesis, and inflammation—essential processes for cellular survival under stress. Elevated expression of HIF-1*α* in pancreatitis indicates its involvement in inflammatory processes [8]. Hypoxia can also activate pancreatic stellate cells (PSCs), which play a crucial role in developing fibrosis and chronic inflammation. Activated PSCs produce extracellular matrix components and inflammatory mediators, contributing to tissue remodeling and fibrosis and further complicating the disease. Under hypoxic conditions, PSC activation can promote *β*-cell death and dysfunction, especially relevant to Type 2 diabetes mellitus, as hypoxia can affect pancreatic islets' function due to increased metabolic demands [9]. In chronic pancreatitis (CP), the persistent hypoxic environment is associated with ongoing inflammation and tissue remodeling, increasing pancreatic cancer risk. Chronic hypoxia is believed to drive HIF-1*α* expression, influencing the tumor microenvironment and promoting tumorigenesis through enhanced angiogenesis and immune evasion [10]. The link between hypoxia and inflammation in CP highlights the necessity of understanding these processes for developing therapeutic strategies that mitigate disease progression and improve patient outcomes. Additionally, hypoxia can affect the expression of inflammatory cytokines and chemokines critical to pancreatitis pathogenesis. For example, hypoxic conditions often lead to the upregulation of interleukin-1*β* (IL-1*β*) and interleukin-6 (IL-6), contributing to the inflammatory environment characteristic of pancreatitis [11]. This inflammatory response can result in systemic complications, including systemic inflammatory response syndrome (SIRS), frequently observed in severe pancreatitis cases. In summary, hypoxia significantly influences pancreatitis pathogenesis, affecting both acute and chronic forms. It drives inflammatory pathways, immune cell recruitment, and tissue injury progression. Understanding the mechanisms by which hypoxia impacts pancreatitis is crucial for developing targeted therapies that could alleviate disease severity and improve patient outcomes. Machine learning algorithms can analyze large medical datasets, including genomic, proteomic, and clinical information, facilitating valuable feature extraction [12–14]. These algorithms automatically detect disease-related patterns, aiding the discovery of novel biomarkers. By employing machine learning techniques, diagnostic accuracy improves significantly, particularly for early stage lesions or rare diseases, by revealing subtle differences that traditional methods may overlook.

The incorporation of machine learning within biomedical research has transformed the way molecular signatures, disease classifications, and predictive models are identified. Conventional statistical methods frequently face challenges when it comes to interpreting intricate, high-dimensional datasets—like transcriptomic, proteomic, or clinical information—because of the nonlinear relationship, noise, and variability that are characteristic of biological systems. In contrast, ML algorithms are highly effective at uncovering hidden patterns from this data, facilitating hypothesis development and speeding up the discovery process. In this study, we focused on developing hypoxia-related genes as diagnostic markers for pancreatitis through the integration of multiple machine learning algorithms. We analyzed the correlation of these genes with immune infiltration and confirmed their differential expression through experimental validation. Our findings underscore the transformative potential of machine learning in pancreatic disease diagnostics, paving the way for improved patient management and targeted treatment strategies.

## 2. Materials and Methods

### 2.1. Sample Collection

This investigation utilized datasets associated with pancreatitis, specifically GSE30134, GSE101462, GSE130563, and GSE149331, to examine the expression levels and functional roles of hypoxia-related genes. The dataset GSE30134 included eight pancreas samples from patients with pancreatitis and nine control samples from individuals without this condition. GSE101462 comprised 10 pancreatitis samples and 3 normal samples for comparison. GSE130563 contained 8 samples from pancreatitis patients and 16 normal control samples, while GSE149331 provided a more extensive cohort with 119 pancreatitis samples.

### 2.2. Weighted Gene Coexpression Network Analysis (WGCNA)

WGCNA was employed to categorize genes based on their expression similarities, facilitating the investigation of connections between different gene clusters and clinical traits. This widely accepted methodology aids in exploring the relationships between clinical characteristics and gene expressions. In this study, emphasis was placed on patients receiving radiotherapy within the pancreatitis cohort. A coexpression network was constructed specifically for the radio-sensitive (RS) and radio-resistant (RR) groups using the R package “WGCNA.” To evaluate gene interactions for adherence to a scale-free distribution, the most suitable soft threshold was identified. Subsequently, a dendrogram illustrating gene adjacency and similarity metrics was developed. To enhance module separation, a dynamic tree cutting algorithm was utilized to merge closely related modules, with each distinct color in the dendrogram representing a unique module of genes exhibiting analogous expression patterns. The association of each gene module with sample characteristics was analyzed through Pearson correlation, leading to the selection of several modules with higher absolute correlation values for further investigation [15, 16].

### 2.3. Functional Analysis

The datasets leveraged during this research originated entirely from the Gene Expression Omnibus (GEO) database and were formatted in MINiML. For specifics regarding the data processing methodologies, please refer to the methods section available on the dataset screening webpage. The differential expression of mRNA was assessed using the limma package in R (Version 3.40.2), with adjusted *p* values analyzed to mitigate the risk of false-positive results. The criteria for identifying differentially expressed mRNA were established as “*p* < 0.05 and log2 (fold change) > 1 or log2(fold change) < −1.” To deepen the understanding of the oncogenic functions associated with the target genes, the clusterProfiler package in R was utilized to analyze the Gene Ontology (GO) functions linked with the identified mRNAs and to enrich the relevant KEGG (Kyoto Encyclopedia of Genes and Genomes) pathways [17, 18].

### 2.4. Analysis of Immune Infiltrates in Pancreatitis Samples

Using the R package GSVA, the single-sample gene set enrichment analysis (ssGSEA) algorithm was employed to assess immune cell infiltration within pancreatitis samples [19, 20]. Heat maps displaying immune infiltration patterns were generated using the ggstatsplot package. Spearman correlation analysis was applied to examine relationships among quantitative variables that did not adhere to normal distribution assumptions. After classifying key genes related to atherosclerosis into high and low expression groups, statistical analyses evaluated the distribution of samples within these categories. Additionally, stacked bar charts were created using the ggplot2 package for data visualization.

### 2.5. Develop a Diagnostic Model

To construct a diagnostic model for atherosclerosis demonstrating reliable accuracy and performance consistency, various machine learning algorithms were employed across multiple configurations. The algorithms included elastic net (Enet), gradient boosting machine (GBM), GlmBoost, least absolute shrinkage and selection operator (LASSO), linear discriminant analysis (LDA), Naive Bayes, plsRglm, random forest (RF), ridge, Stepglm, support vector machine (SVM), and extreme gradient boosting (XGBoost). Model training was conducted using the GSE30134, GSE101462, and GSE130563 datasets, and validation was performed with the GSE149331 dataset. Each combination of algorithms was evaluated based on the area under the curve (AUC) metric, with the configuration yielding the highest average AUC selected as the optimal model.

### 2.6. Quantitative Real-Time Polymerase Chain Reaction (qRT-PCR)

Total RNA was extracted from tissues using TRIzol reagent (Invitrogen). FastKing RT SuperMix (Tiangen, Beijing, China) was employed for reverse transcription. Next, RT-RCR was carried out on the CFX Connect RT-PCR detection system (Bio-Rad, CA, United States) through SYBR Green PCR Master Mix (Thermo Fisher Scientific, MA, United States). The 2^−*ΔΔ*CT^ method was used to quantify gene expression with GAPDH as the internal control. The primer pairs corresponding to the target genes included the following: GAPDH (forward: CGGAGTCAACGGATTTGGTCGTAT, reverse: AGCCTTCTCCATGGTGGTGAAGAC), RAP1GDS1 (forward: TGGCATCAAGCATCTAGTT, reverse: GCGAAGTTTGGATACGACA), TOP2A (forward: TCCTGCCTGTTTAGTCGCTT, reverse: ATTTACAGGCTGCAATGGTGAC), ADK (forward: AGAGGCAGCGAATCGTGATCTTCA, reverse: ACCTCCAACAAATGCATCTCCAGC), POLL (forward: CATCAAAAGTACTTGCAAAGATTCC, reverse: GGGAGCTCAGCCACTCTTC), CD44 (forward: TGACACTGTCCAAAGGTTTTC, reverse: TCACTAATAGGGCCAGCCTC), and CD4 (forward: TCGGATTGACTGCCAACTCTG, reverse: AAGGCGAGCGGGAAGGAGAA).

### 2.7. ELISA Assay

The levels of IL-6, IL-18, and IL-1*β* in pancreatitis samples were determined using ELISA kits according to the described protocol. Briefly, samples were incubated consecutively with antibody-coated plates, biotinylated antibodies, streptavidin antibodies, substrate solution, and stop solution as indicated in the protocol. Subsequently, the optical density at 450 nm was measured using a microplate reader. All samples were tested in triplicate.

### 2.8. Statistical Analyses

Statistical differences between the two groups were examined using a *t*-test. Pearson's method was utilized for correlation assessments, with a significance threshold (*p* value) set at < 0.05, indicating statistically significant findings.

## 3. Results

### 3.1. Identification of Key Hypoxia-Related Regulatory Genes in Pancreatitis

**Figure 1:**
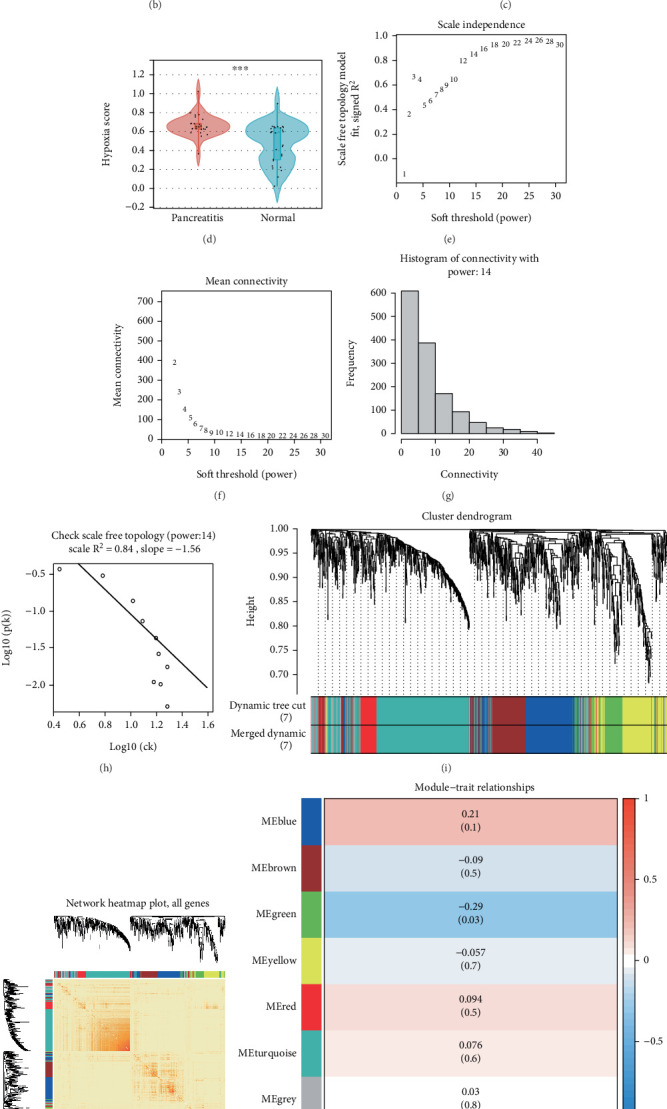
WGCNA identifies hypoxia-related genes. (a) The standardized data is presented in the form of a box plot. (b) PCA results prior to the elimination of batch effects across various datasets. (c) PCA results following the removal of batch effects between the distinct datasets. (d) Analysis of the differences in hypoxia scores among various groups. (e–h) The optimal power for soft thresholding was established to be 14. (i, j) A weighted coexpression network was constructed using the selected power values. (k) A heat map was created to show the correlations among trait modules. (l) Diagram depicting the interaction network of genes related to hypoxia.

To identify biomarkers linked to the progression of pancreatitis, we acquired data from three distinct pancreatitis datasets (GSE30134, GSE101462, and GSE130563) housed within the GEO database, incorporating a total of 26 samples from pancreatitis patients and 28 samples from healthy controls. Initially, we normalized and integrated these high-throughput sequencing datasets into a comprehensive collective for subsequent analysis. We then performed principal component analysis (PCA), displaying results prior to and postbatch removal, which evidenced effective merging of datasets for further examination (Figures 1a, 1b, and 1c). Utilizing the HALLMARK_HYPOXIA gene set and applying the ssGSEA method, we calculated hypoxia scores, determining that patient with pancreatitis exhibited significantly elevated hypoxia scores compared to controls (Figure 1d). Samples were stratified into high and low hypoxia score groups based on these calculated scores. To discover hypoxia-related genes potentially influential in pancreatitis, we executed WGCNA on the integrated dataset. To confirm adherence to a scale-free topology, we adjusted the weight settings of the adjacency matrix and determined an optimal power value of 14 (Figures 1e, 1f, 1g, and 1h). Consequently, we established a weighted coexpression network classifying all genes into seven distinct modules (Figure 1i,j). Employing the Pearson correlation method, we assessed the correlation coefficients and associated *p* values between module eigengenes and specified traits, revealing that the green module displayed the most substantial association with a coefficient of −0.29 (Figure 1k). We subsequently illustrated the correlation network among the top 50 genes within the green module (Figure 1l).

### 3.2. Identification of Key Hypoxia Genes Using Machine Learning Algorithms

**Figure 2:**
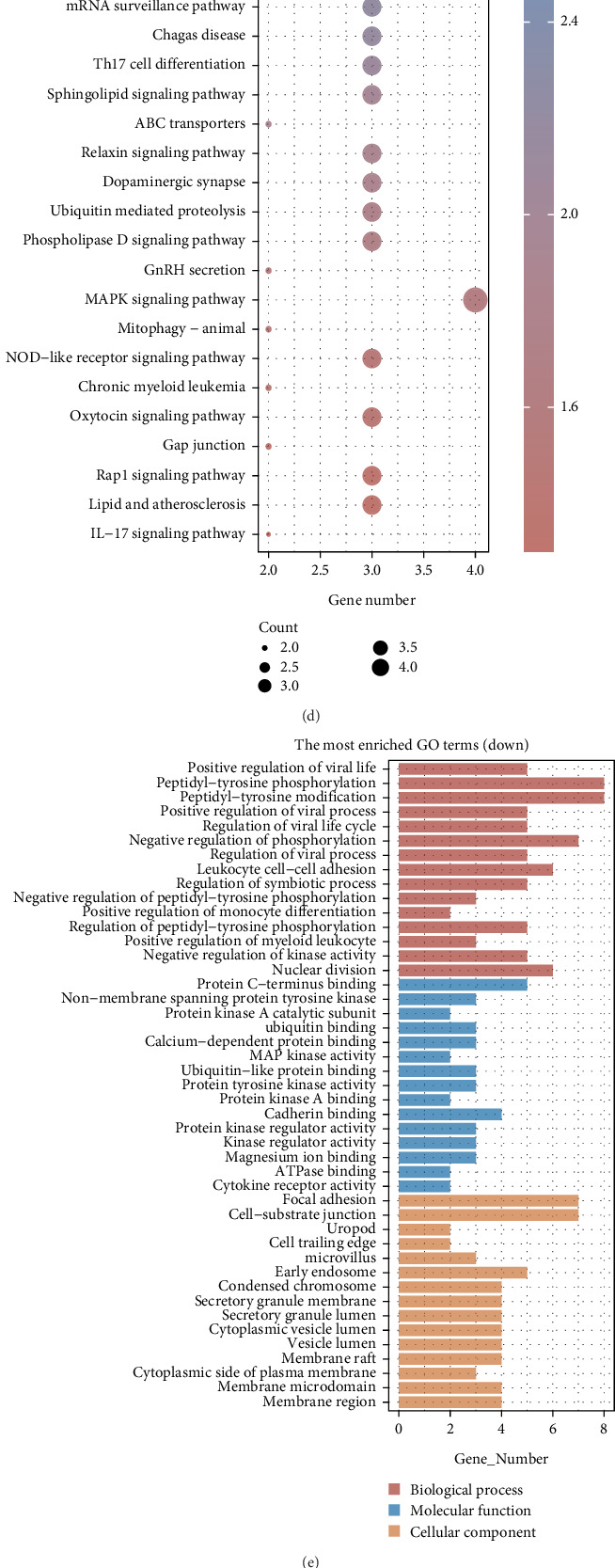
Six hypoxia-related genes identified as key genes for pancreatitis. (a, b) Analysis of differences. (c–f) Functional analysis. (g–i) The identification of pancreatitis-related hypoxia genes is achieved using XGBoost and random forest algorithms.

In analyzing datasets GSE30134, GSE101462, and GSE130563, we categorized samples associated with pancreatitis against normal controls. Employing criteria of *p* < 0.05 and log2(fold change) thresholds of greater than 1 or less than −1, we identified 53 genes, comprising 41 upregulated and 12 downregulated genes (Figure 2a,b). To further elucidate the functional roles of these candidate genes, we undertook functional enrichment analyses. GO provided insight into annotating gene functions across molecular function (MF), biological process (BP), and cellular component (CC). Simultaneously, KEGG pathway enrichment analysis revealed that the upregulated genes were significantly linked to pathways such as MAPK, TGF-beta signaling, Th17 cell differentiation, oxytocin signaling, and Rap1 signaling pathways. The GO analyses indicated that these genes were predominantly involved in activities related to membrane docking, ATPase functions, and basolateral plasma membrane structures (Figure 2c,d). Conversely, downregulated genes were associated with pathways linked to human T-cell leukemia Virus 1, cell cycle regulation, Th1 and Th2 cell differentiations, T-cell receptor signaling, and PD-L1/PD-1 interactions. GO analyses for these genes revealed associations with processes like peptidyl-tyrosine phosphorylation, protein C-terminus binding, and focal adhesion (Figure 2e,f). Merging the top 50 genes identified from WGCNA with those discovered through differential expression analysis yielded 23 key differential genes (Figure 2g). Utilizing XGBoost and RF, we pinpointed 10 genes closely related to pancreatic cancer (Figure 2h,i), culminating in the identification of RAP1GDS1, TOP2A, ADK, POLL, CD44, and CD4 as pivotal genes linked to hypoxia in pancreatitis.

### 3.3. Expression Analysis of Hypoxia-Related Genes

**Figure 3:**
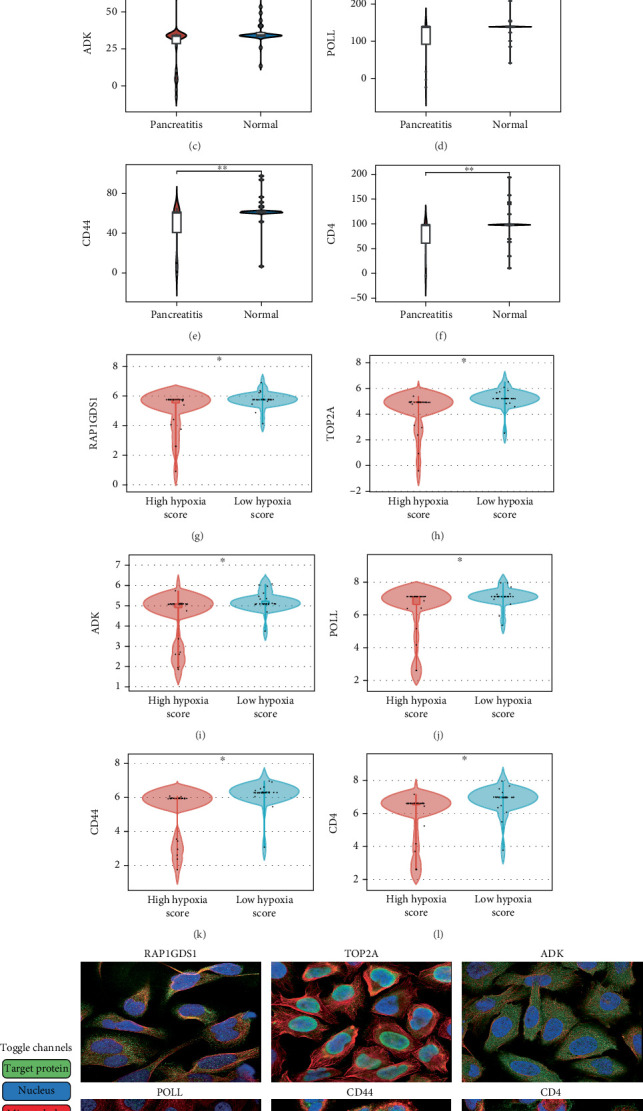
Six hypoxia-related genes are all lowly expressed in pancreatitis. (a–f) Expression analysis of hypoxia-related genes. (g–l) Expression differences of six hypoxia-related genes in different hypoxia score groups. (m) Localization analysis of APLNR, PCDH12, PODXL, SLC40A1, TM4SF18, and TNFRSF25 in cells. ⁣^∗^*p* < 0.05.

An investigation into the expression levels of RAP1GDS1, TOP2A, ADK, POLL, CD44, and CD4 was carried out utilizing datasets GSE30134, GSE101462, and GSE130563. The analyses revealed significant upregulation of these genes in normal samples relative to those affected by pancreatitis (Figures 3a, 3b, 3c, 3d, 3e, and 3f). Further examination of expression dynamics within samples categorized by high and low hypoxia scores highlighted substantial expression differences, indicating elevated levels in the medium hypoxia score cohort (Figures 3g, 3h, 3i, 3j, 3k, and 3l). The Human Protein Atlas was consulted to determine gene localization, establishing that RAP1GDS1 and CD44 were predominantly found in the cytosol; TOP2A, ADK, and POLL primarily localized in the nucleoplasm; and CD4 associated with the plasma membrane (Figure 3m).

### 3.4. Construction of a Diagnostic Model for Pancreatitis

**Figure 4:**
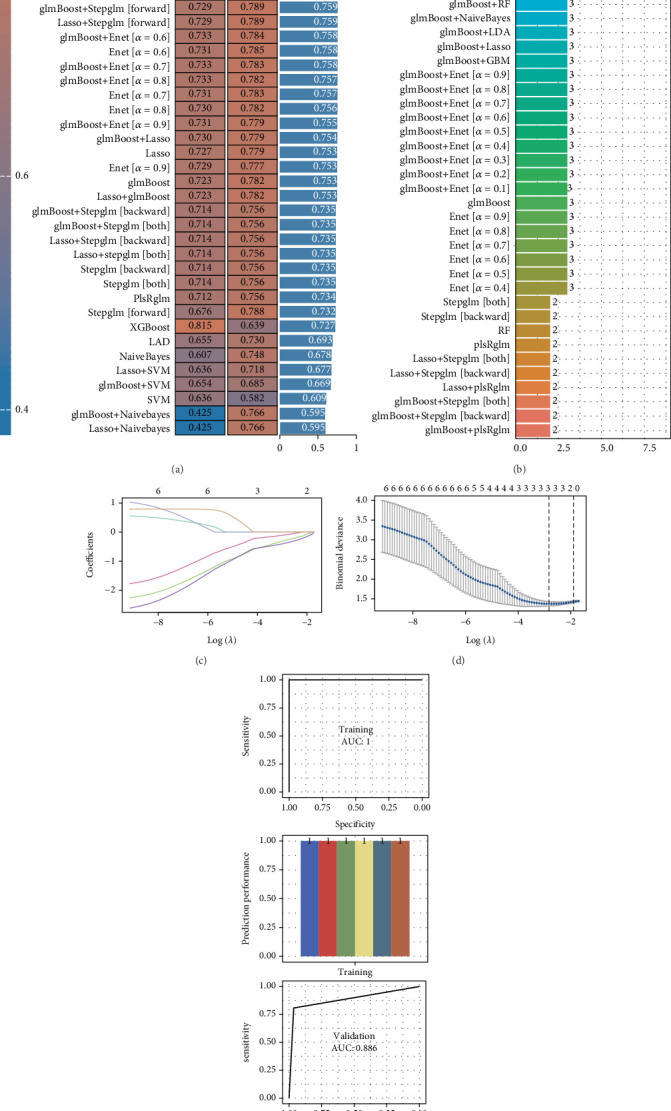
LASSO + XGBoost algorithm builds diagnostic model. (a) AUC values of different algorithm combinations. (b) Number of genes included in different algorithm combinations. (c, d) LASSO algorithm for screening diagnostic genes. (e) Verification of diagnostic model constructed by XGBoost algorithm.

The utilization of machine learning technology is experiencing rising relevance in biomedicine, facilitating the integration and analysis of clinical data to inform physician decision-making and individualized treatment strategies. In the present study, we constructed a diagnostic model for pancreatitis aimed at enhancing early disease detection. The model was developed using datasets GSE30134, GSE101462, and GSE130563, with validation performed on GSE149331. Among the numerous algorithmic combinations examined, LASSO combined with XGBoost exhibited peak effectiveness for model development. A comprehensive overview of the gene counts integrated into each algorithmic combination is presented for clarity (Figure 4a,b). The AUC assessment indicated values of 0.945 for the training datasets and 0.851 for the validation dataset. Following identification of three crucial genes using LASSO, a diagnostic model was established via the XGBoost algorithm, further substantiating our investigative conclusions (Figures 4c, 4d, and 4e).

### 3.5. Functional Analysis of Hypoxia-Related Genes

**Figure 5:**
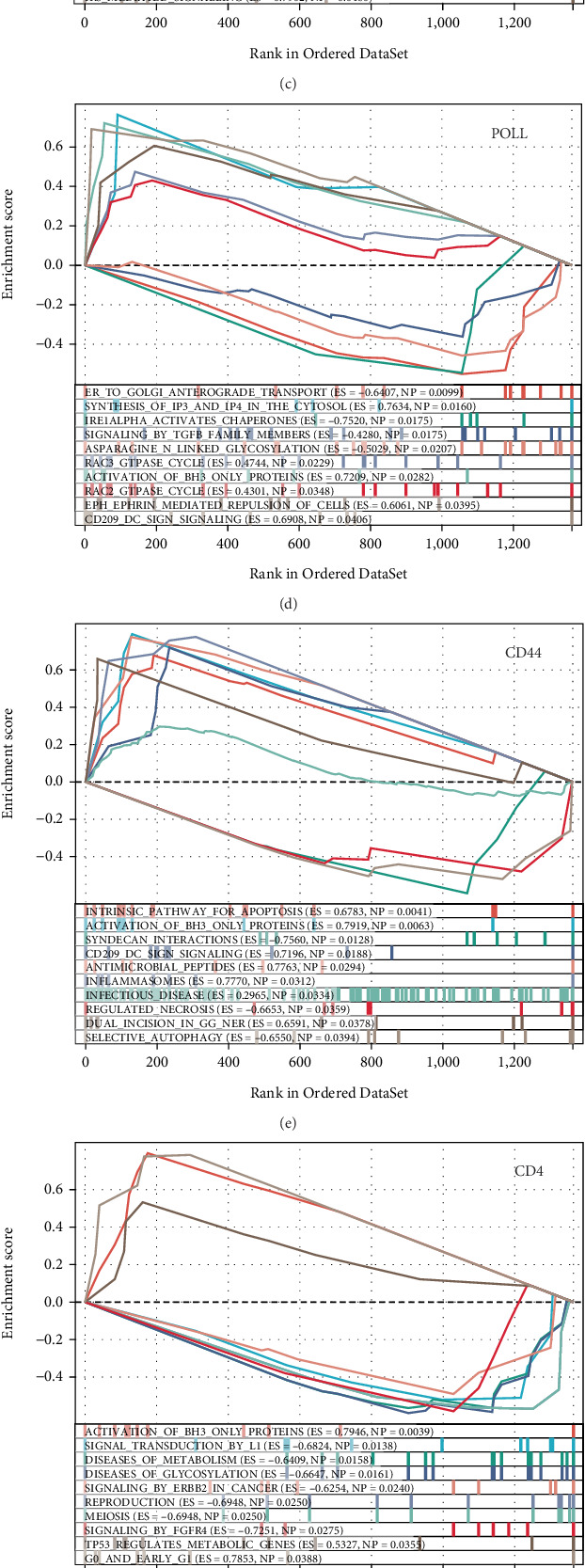
Functional analysis of genes. (a–f) GSEA for RAP1GDS1, TOP2A, ADK, POLL, CD44, and CD4. (g, h) GenDoma database analysis of gene interaction network diagram.

To elucidate the potential functions of the identified hypoxia-related genes in pancreatitis, we conducted a detailed functional analysis. Our findings indicate that RAP1GDS1 is involved in rRNA modification in both the nucleus and cytosol, along with constitutive signaling by EGFRVIII and metabolic diseases (Figure 5a). TOP2A is associated with infectious diseases, cellular responses to starvation, and selenoamino acid metabolism (Figure 5b). ADK plays a role in mitotic prometaphase, visual phototransduction, and WNT signaling pathways (Figure 5c). POLL is linked to TGF-beta family signaling, the RAC3 GTPase cycle, and CD209 DC-SIGN signaling (Figure 5d). CD44 is implicated in the intrinsic pathway of apoptosis, syndecan interactions, and selective autophagy processes (Figure 5e). Lastly, CD4 is connected to glycosylation disorders, reproductive processes, and FGFR4 signaling (Figure 5f). Additionally, we analyzed the interacting proteins, associated transcription factors, relevant miRNAs, and potential therapeutic drugs via the GenDoma database (Figure 5g,h).

### 3.6. Analysis of Immune Cell Infiltration

**Figure 6:**
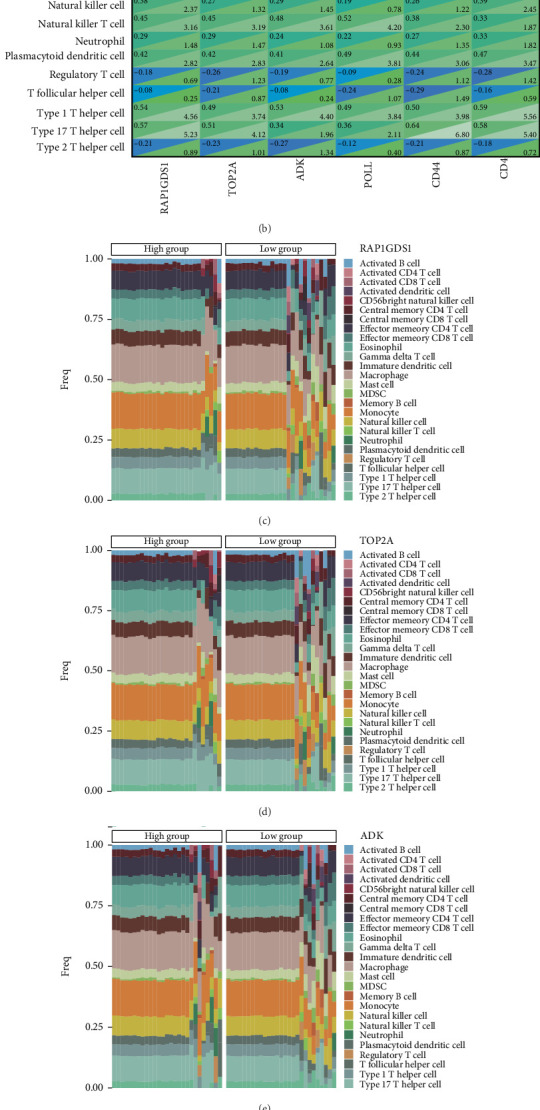
The connection between essential identified genes and immune cell populations. (a) A heat map illustrating the relationships among 28 distinct types of immune cells. (b) A heat map showcasing the connections of RAP1GDS1, TOP2A, ADK, POLL, CD44, and CD4 with these 28 immune cell types, where correlation coefficients are displayed in the upper left section and *p* values are found in the lower right section. (c–h) The proportion of immune cell infiltration per sample is represented according to the classification based on the identified genes. -*p* > 0.05, ⁣^∗^*p* < 0.05, ⁣^∗∗^*p* < 0.01, ⁣^∗∗∗^*p* < 0.001.

Recent studies indicate that immune cell infiltration plays a crucial role in the progression of pancreatitis. In our investigation, we analyzed the correlation between the characteristics of pancreatitis and immune cell infiltration. Following the work of Pornpimol et al., who identified 28 genes as markers for various immune cells, we utilized ssGSEA to evaluate immune cell scores in patients based on this specific gene set. Heat maps were subsequently generated to visualize the infiltration levels of 28 distinct immune cell types, revealing a positive correlation in the infiltration levels of most immune cells (Figure 6a). Further analysis involved examining the relationship between the genes RAP1GDS1, TOP2A, ADK, POLL, CD44, and CD4 and the immune cell types. This analysis resulted in additional heat maps (Figure 6b), which highlighted that RAP1GDS1 expression was significantly associated with the infiltration of activated B cells and activated dendritic cells. Additionally, TOP2A showed a strong correlation with activated B-cell infiltration. Meanwhile, ADK expression was notably linked to the infiltration levels of both activated B cells and activated CD8 T cells. The expressions of POLL, CD44, and CD4 were found to be closely associated with the infiltration of central memory CD4 T cells. To further investigate the samples, we categorized them based on the expression levels of the aforementioned genes. We created heat maps to illustrate the abundance of immune cells across the samples, classifying them into high and low expression groups, and used distinct color codes to represent various immune cell types (Figures 6c, 6d, 6e, 6f, 6g, and 6h).

### 3.7. Molecular Docking of Key Hypoxia-Related Genes and Therapeutic Drugs

**Figure 7:**
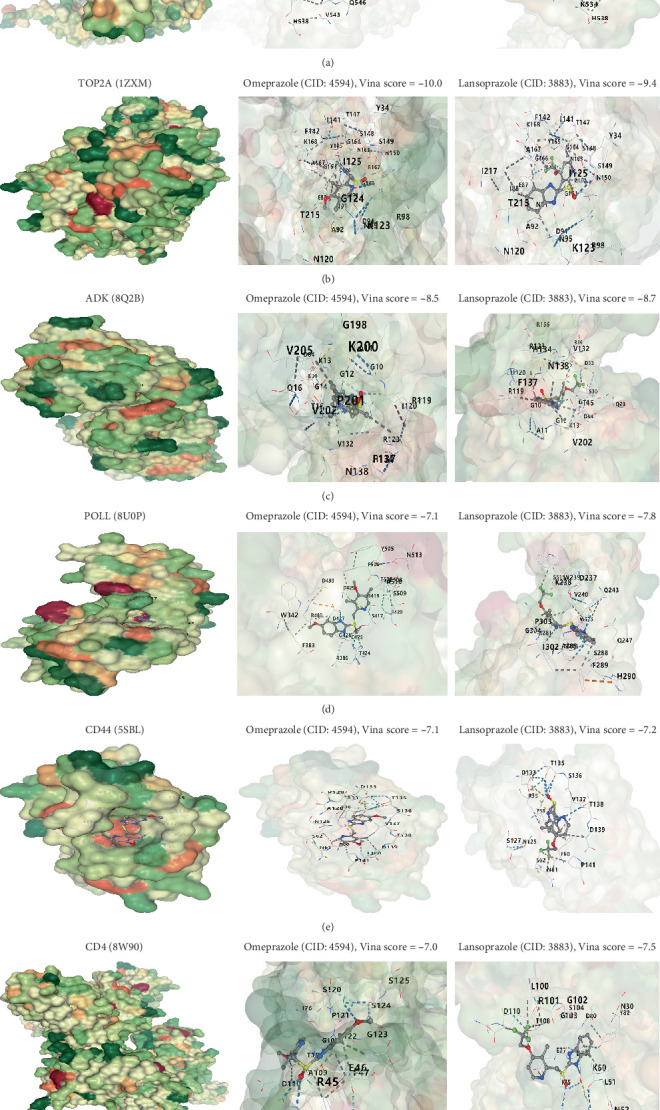
All six proteins exhibited strong binding affinity to both omeprazole and lansoprazole. (a–f) Molecular docking analysis.

In the context of treating pancreatitis, drugs primarily targeting gastric acid secretion, such as omeprazole and lansoprazole, are commonly employed. In our study, we evaluated the binding affinities of the genes RAP1GDS1, TOP2A, ADK, POLL, CD44, and CD4 with these two medications through molecular docking analysis. Our findings demonstrated that all six proteins exhibited strong binding affinities for both omeprazole and lansoprazole. Notably, the Vina scores for RAP1GDS1 were −7.6 with omeprazole and −8.0 with lansoprazole (Figure 7a). TOP2A showed scores of −10.0 and −9.4, respectively (Figure 7b). For ADK, the Vina scores were −8.5 for omeprazole and −8.7 for lansoprazole (Figure 7c). POLL had scores of −7.1 and −7.8 (Figure 7d), and CD44 scored −7.1 and −7.2 (Figure 7e), while CD4 recorded Vina scores of −7.0 with omeprazole and −7.5 with lansoprazole (Figure 7f).

### 3.8. Expression Verification of Key Hypoxia-Related Genes

**Figure 8:**
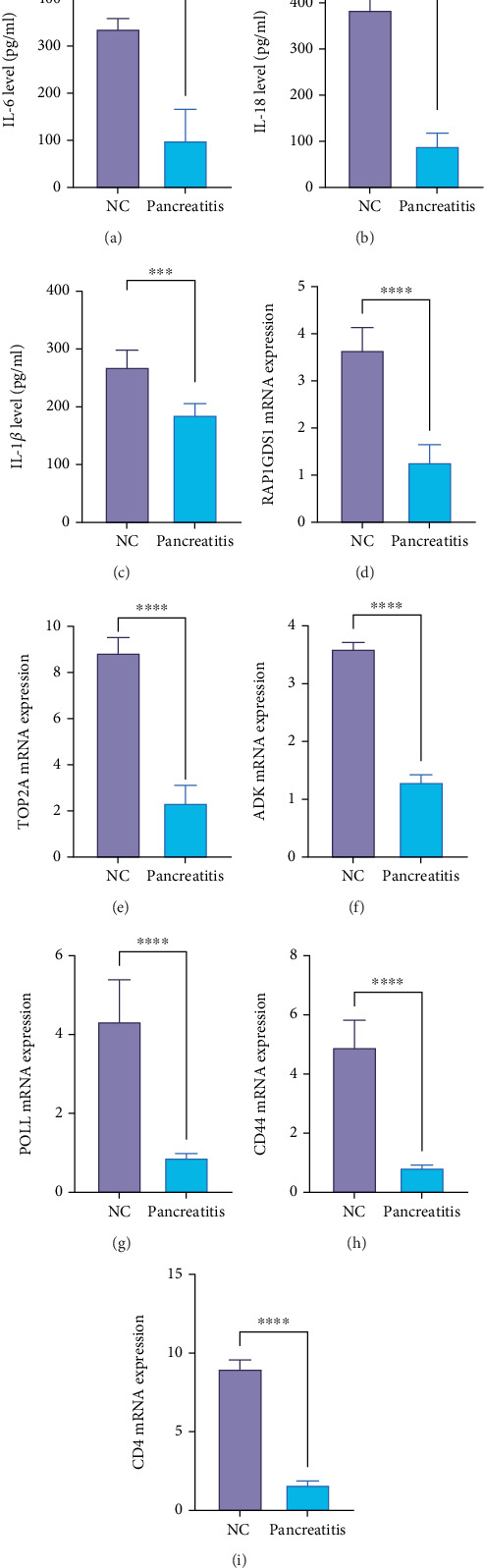
Hypoxia-related genes are underexpressed in pancreatitis. (a–c) Elisa detects the expression of inflammatory factors. (d–i) Expression analysis of hypoxia-related genes in pancreatitis. ⁣^∗∗∗^*p* < 0.001, ⁣^∗∗∗∗^*p* < 0.0001.

To further validate the roles of key hypoxia-related genes in pancreatitis, we first measured the expression levels of inflammatory factors in serum samples using an ELISA. Results indicated that IL-6, IL-18, and IL-1*β* were all significantly elevated in pancreatitis samples (Figures 8a, 8b, and 8c). We then conducted qRT-PCR on serum samples from five pancreatitis patients and five healthy controls to examine the expression levels of RAP1GDS1, TOP2A, ADK, POLL, CD44, and CD4. The results revealed that these genes were significantly downregulated in patients with pancreatitis compared to the normal controls (Figures 8d, 8e, 8f, 8g, 8h, and 8i). This underscores the potential significance of these key hypoxia-related genes in the pathophysiology of pancreatitis.

## 4. Discussion

Identifying reliable diagnostic markers for pancreatitis is essential due to the complex and variable nature of the disease, which can manifest as both acute and chronic forms. AP is characterized by a rapid onset of inflammation in the pancreas, often leading to serious complications and a high mortality risk if not diagnosed and managed promptly. Timely detection of AP is crucial for effective treatment, significantly improving patient outcomes. Currently, diagnosis largely relies on clinical symptoms, imaging techniques, and serum enzyme levels, particularly amylase and lipase. However, these conventional markers have limitations, including false negatives and challenges in differentiating between various pancreatic conditions such as pancreatitis and pancreatic ductal adenocarcinoma (PDAC) [21, 22].

Recent research underscores the urgent need for more accurate and reliable biomarkers for early pancreatitis detection. For example, one study identified 239 potential metabolic biomarkers for diagnosing CP and its acute exacerbations, highlighting the importance of serum metabolic profiles in distinguishing CP patients from those with nonpancreatic conditions [23]. These findings suggest that metabolic profiling could enhance diagnostic precision and provide a deeper understanding of the disease. Additionally, the role of inflammatory markers in pancreatitis has gained attention. Elevated levels of tumor necrosis factor-alpha (TNF-*α*) and IL-1*β* have been associated with the severity of inflammation in AP, indicating their potential as diagnostic markers [24]. Furthermore, the pancreatic renin–angiotensin system has been implicated in AP development, suggesting that targeting this pathway may lead to new biomarkers for both diagnosis and treatment [25]. In summary, the pressing need for diagnostic markers in pancreatitis is emphasized by the shortcomings of existing techniques and the disease's intricate nature. Advances in metabolomics, inflammatory markers, gut microbiota profiling, and molecular indicators such as microRNAs hold promise for improving diagnostic accuracy and patient management. Continued research in these areas is vital for developing reliable, noninvasive diagnostic solutions that enable early detection and treatment of pancreatitis, ultimately enhancing patient outcomes and reducing morbidity and mortality associated with the condition.

Under low oxygen conditions, pancreatic tissue damage results from oxidative stress, inflammation, and the activation of PSCs. Hypoxia, defined as reduced oxygen supply, can lead to significant cellular dysfunction and death. A key factor contributing to pancreatic damage during hypoxic episodes is the increased production of reactive oxygen species (ROS). In hypoxic environments, pancreatic *β*-cells experience elevated oxidative stress, negatively impacting their survival. The ROS generated by hypoxia can initiate various cell death pathways, leading to either apoptosis or necrosis of pancreatic cells. For instance, in *β*-cells, hypoxia can cause ROS accumulation, activating inflammatory responses and cell death mechanisms, further exacerbating tissue injury [26].

The hypoxic state can also instigate inflammatory responses within pancreatic tissue, as the release of proinflammatory cytokines and the activation of immune cells contribute to an overall inflammatory environment that aggravates tissue damage. Inflammatory mediators can activate PSCs and promote oxidative stress, creating a detrimental cycle. Additionally, hypoxia can induce mitochondrial dysfunction, critical for energy production in pancreatic cells. Impaired mitochondrial function results in decreased ATP production and increased ROS generation, exacerbating cellular damage. Mitochondrial permeability transition pores (mPTPs) might play a significant role in this process; their opening can cause the release of proapoptotic factors, intensifying cell death [27, 28]. In conclusion, damage to pancreatic tissue in hypoxic conditions involves a complex interplay of oxidative stress, inflammation, and mitochondrial dysfunction. Understanding these underlying processes is crucial for developing therapeutic strategies aimed at protecting pancreatic tissue from hypoxic injury.

Multiomics analysis has been extensively employed in identifying diagnostic and therapeutic markers for tumors and inflammation-related diseases, particularly those targets associated with immune responses. However, conclusions derived solely from transcriptomic data obtained through multiomics analysis exhibit certain limitations, thereby necessitating in vivo and in vitro experimental analyses [29–31]. This study employed rigorous bioinformatics methods to identify hypoxia-related regulatory genes and immune infiltration patterns in pancreatitis; however, it is essential to acknowledge certain limitations. Firstly, the sample size used in this study is relatively small, which may introduce bias and reduce the statistical power of the differential expression analysis. Future research with larger and more balanced cohorts is necessary to validate these findings. Secondly, although bioinformatics tools such as WGCNA and ssGSEA provide valuable insights, they rely on computational inference rather than direct experimental validation. Therefore, functional studies in cellular or animal models are required to confirm the mechanistic roles of the identified genes in the progression of pancreatitis under hypoxic conditions. Addressing these limitations in future work will enhance the translational relevance of our findings.

In our study, we identified key hypoxia-related genes—RAP1GDS1, TOP2A, ADK, POLL, CD44, and CD4—in pancreatitis samples using methods such as WGCNA, XGBoost, and RF algorithms. We experimentally confirmed that these genes are expressed at significantly lower levels in pancreatitis samples, reinforcing their potential role as biomarkers for the disease. This finding opens avenues for future research into targeted therapies and diagnostic tools that can effectively manage pancreatitis.

## 5. Conclusion

In summary, our study underscores the significance of hypoxia-related genes in understanding the pathophysiology of pancreatitis and opens new avenues for research aimed at leveraging these markers for diagnostic and therapeutic purposes.

## Data Availability

The data that support the findings of this study are available from the corresponding author upon reasonable request.
